# Combined therapeutic strategy based on blocking the deleterious effects of AGEs for accelerating diabetic wound healing

**DOI:** 10.1093/rb/rbae062

**Published:** 2024-06-05

**Authors:** Yang Yang, Siwen Huang, Qing Ma, Ning Li, Runchu Li, Yongjun Wang, Hongzhuo Liu

**Affiliations:** Department of Pharmaceutics, College of Pharmacy, Shenyang Pharmaceutical University, Shenyang 110016, China; Department of Pharmaceutics, Wuya Collage of Innovation, Shenyang Pharmaceutical University, Shenyang 110016, China; Department of Pharmaceutics, Wuya Collage of Innovation, Shenyang Pharmaceutical University, Shenyang 110016, China; Department of Pharmaceutics, Wuya Collage of Innovation, Shenyang Pharmaceutical University, Shenyang 110016, China; Beijing No. 4 High School International Campus, Beijing 100031, China; Department of Pharmaceutics, Wuya Collage of Innovation, Shenyang Pharmaceutical University, Shenyang 110016, China; Department of Pharmaceutics, Wuya Collage of Innovation, Shenyang Pharmaceutical University, Shenyang 110016, China

**Keywords:** advanced glycation end products, chronic skin wounds, combined therapeutic strategy, NPs/hydrogel composite dressings, localized co-delivery

## Abstract

Diabetic foot ulcer is a serious complication of diabetes. Excessive accumulation of advanced glycation end products (AGEs) is one of the critical pathogenic factors in postponing diabetic wound healing. The main pathogenic mechanisms of AGEs include inducing cellular dysfunction, prolonging inflammatory response, increasing oxidative stress and reducing endogenous nitric oxide (NO) production. Combination therapy of blocking the deleterious effects of AGEs and supplementing exogenous NO is hypothesized to promote diabetic wound healing. Here, we presented nanoparticles/hydrogel composite dressings to co-delivery rosiglitazone and S-nitroso glutathione into the wound bed. The designed co-delivery system augmented the survival of fibroblasts, reduced oxidative stress levels, reversed the change of mitochondrial membrane potential and decreased the proinflammatory cytokine expression. Local sustained release of therapeutic agents significantly improved the wound healing of diabetic rats including increasing the wound closure rate, alleviating inflammation, promoting collagen fiber production and angiogenesis. Our finding indicated this local deliver strategy aimed at inhibiting the toxic effects of AGEs has great clinical potential for diabetic wound treatment.

## Graphical abstract

**Figure:**
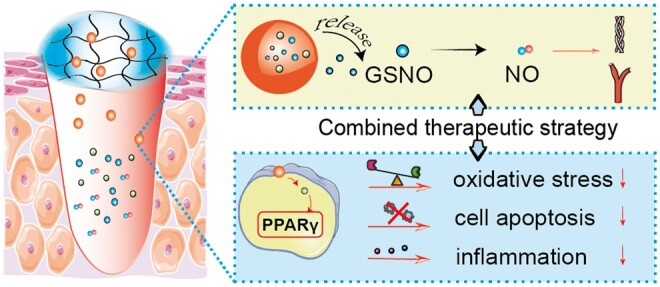

## Introduction

Diabetic foot ulcers (DFU) characterized with chronic and nonhealing wounds are one of the serious complications of diabetes. Current therapeutic strategies such as wound debridement [1], infection control [2], hyperbaric oxygen treatment [3] and cell therapy [4, 5] still remain poor therapeutic effects and high recurrence rate. The pathophysiological process of DFU is complicated and affected by multi-factors like the toxic effects of advanced glycation end products (AGEs) [6], persistent hypoxia state [7] and abnormal immune microenvironments [8]. AGEs are heterogeneous molecules generated by sugars interacting with proteins or lipids via non-enzymatic glycation. Excessive accumulation of AGEs induced by hyperglycemia resulted in the prolonged expression of proinflammatory cytokines, oxidative stress derived damage [9] and extracellular matrix (ECM) changes [10]. More importantly, AGEs inhibit the expression of nitric oxide (NO) synthase in endothelial cells, hampering angiogenesis and re-epithelialization during wound healing [11]. The impaired vascular adaptation are hallmarks in DFU, stimulating the abnormal immune cascade and eventually preventing the transition of wounds from the inflammatory to the proliferative phase. Therefore, AGEs may become an important target for DFU therapy.

Many attempts have been made by designing different strategies to inhibit AGEs. One strategy emphasis on hindering AGEs/RAGE interactions with RAGE antagonists. SRAGE, a competitive inhibitor of AGEs, which has been proved to accelerate the wound healing with 90% wound closure on post-wounding Day 28 [12]. However, its application was limited due to the rapid degradation of sRAGE in the highly proteolytic wound environment. Another strategy highlights the investigations of anti-AGEs agents [13]. An outstanding example is rosiglitazone (RGZ), a PPARγ agonist, has been proven to block the deleterious effects of AGEs by attenuating dysfunction of endothelial cells [14] and reducing AGE-induced ROS generation [15]. However, the use of RGZ is hindered by their severe adverse effects. The oral administration of RGZ exists cardiovascular risks including fluid retention, heart failure and increased levels of low-density lipoprotein cholesterol, which makes it been highly restricted access by FDA since 2010 [16]. Therefore, local delivery of RGZ to the wound bed is necessary to provide an effective and safe treatment.

NO, as an endogenous gas transmitter, plays multiply regulatory roles in wound healing such as wide-ranging antibacterial activity, cell proliferation, collagen formation and angiogenesis [17–19]. Based on the hindrance of AGEs on endogenous NO generation, supplementing exogenous NO will have a positive promoting effect on the healing of chronic wounds in diabetes [20]. S-nitrosoglutathione (GSNO) is the most intensively investigated exogenous NO donor due to its easy synthesis and high safety [21]. Many strategies for delivering GSNO have been proven to exhibit multiple effects such as antibacterial activity [22, 23] and promoting angiogenesis [24]. Given the benefits of GSNO in promoting wound healing, a combinatorial therapy by co-delivering RGZ and GSNO will be expected to further enhance the wound healing.

Nanoparticles (NPs) are widely utilized as a co-deliver carrier by virtue of their high surface areas, adjustable drug loading and release kinetics and variable size and shape [25–27]. Recently, NPs have gradually been applied for wound treatment and show excellent performance in the field of bacterial inhibition [28, 29], anti-inflammation [30] and promoting cell proliferation [31]. Despite having many advantages, using a single NPs as a wound dressings still faces many challenges. For example, it is difficult to be fixed at the wound site when injected *in situ*. NPs also lack the ability to seal wounds, accomplish hemostasis and maintain a moist environment [32]. Thus, producing wound dressings by loading NPs into hydrogel can remedy these shortcomings and enhance the efficacy of NPs.

**Scheme 1.**
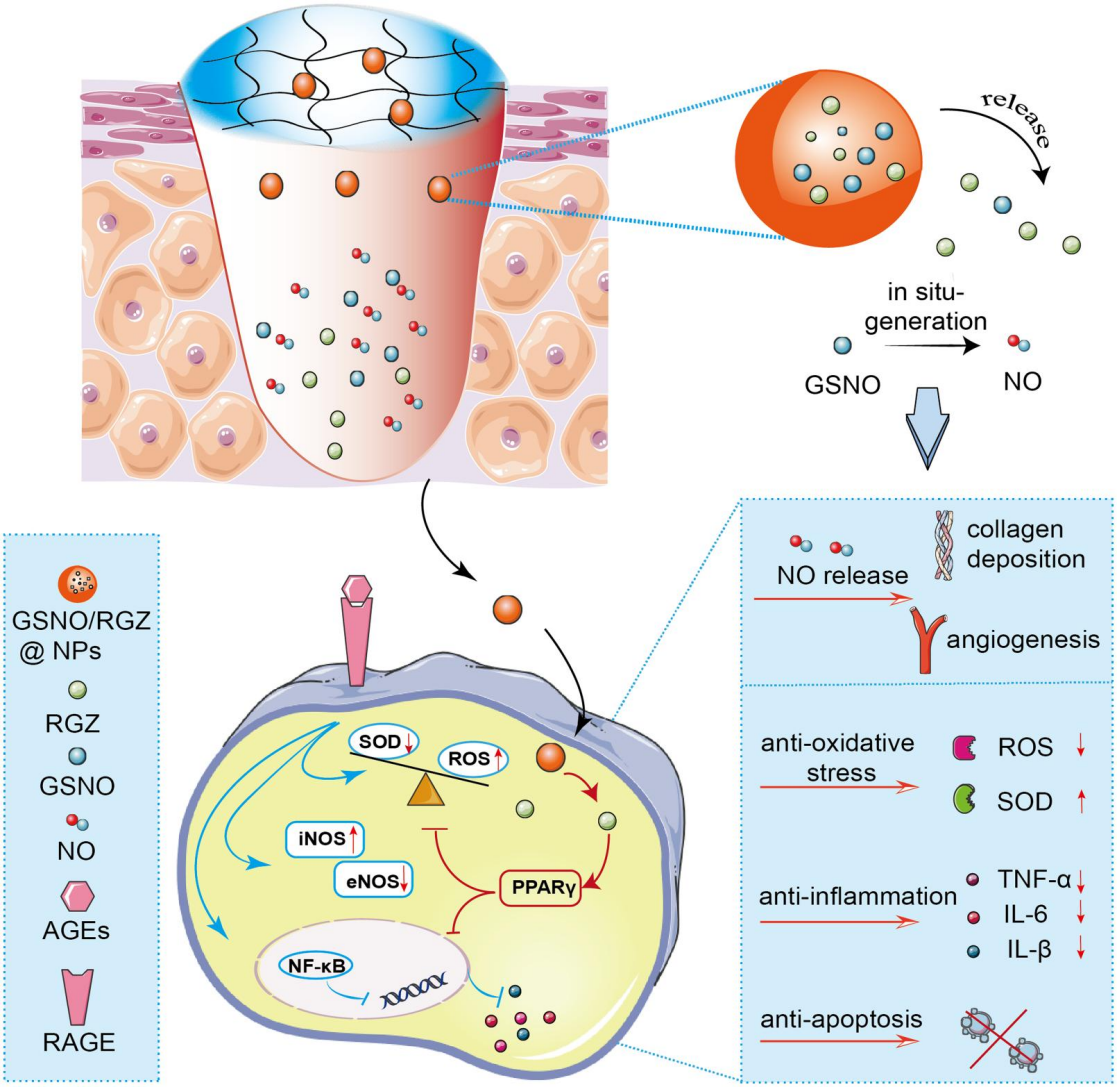
Schematic illustration of NPs/hydrogel locally co-delivery system. The co-delivery system reverses AGE-induced deleterious effects by local supplementation of NO and intracellular delivery of RGZ.

Here, we presented a localized delivery system based on GSNO and RGZ co-loaded NPs/hydrogel composite dressings. Local delivery through hydrogel dressings permitted a release of the cargo at wound bed, thus limiting the risk of side effects. Therapeutic agents were efficiently delivered to the cells through phagocytosis of the NPs by the cells, as shown in Scheme 1. In wound micoenvironment, activation of PPARγ by RGZ inhibited AGE induced oxidative stress, inflammation and apoptosis. Topical NO supplementation helps collagen deposition and angiogenesis at the wound site. We expect that this combined therapeutic strategy may offer a new treatment option for diabetic wound healing.

## Materials and methods

### Materials

Eudragit@RL PO were purchased from Chineway Pharmaceutical Technology Co., Ltd (Shanghai, China). Poloxamer188 were provided by Fengli Pharmaceutical Technology Co., Ltd (Beijing, China). GSNO were purchased from Macklin Bio-Technology Co., Ltd (Shanghai, China). RGZ were purchased from Yuanye Bio-Technology Co., Ltd (Shanghai, China). Pluronic F127 were purchased from Rionlon Pharmaceutical Technology Ltd (Shanghai, China). All assay kits were provided by Neobioscience Bio-Technology Co., Ltd (Shenzheng, China) and Meilunbio Bio-Technology Co., Ltd (Dialian, china).

### Synthesis of GSNO

GSNO was synthesized by one-step nitrosation reaction [33]. Specifically, glutathione (GSH) was dissolved in hydrochloric acid, followed by the addition of the equimolar sodium nitrite (NaNO_2_). The mixed solution was stirred continuously for 40 min in an ice bath. After then, the precipitation was obtained in cold acetone and vacuum-filtered. The product was washed with cold water and cold acetone for three times, freeze-dried and stored in dark at −20°C. The structure of GSNO was verified by ^1^H NMR (400M, AV400/600, Bruker, America) and the result was shown in Figure S1.

### Preparation of GSNO/RGZ@NPs

The combination drugs loaded NPs were prepared using double emulsion-solvent evaporation method. Briefly, 200 μl of 0.1% (w/v) poloxamer 188 solution was added dropwise to 2 ml of Eudragit@RL PO (200 mg) in dichloromethane and the mixture was sonicated for 4 min. The prepared first phase was added to 8 ml of 0.1% (w/v) poloxamer 188 solution and allowed to sonication and solidify at room temperature. Afterwards, the NPs suspension was centrifuged and the precipitation was redispersed in 10 ml of 10% trehalose solution and freeze-dried. To upload drugs in NPs, GSNO and RGZ were included in 200 μl of poloxamer 188 solution and 2 ml of Eudragit@RL PO in dichloromethane respectively. The obtained GSNO/RGZ@ NPs was stored in dark at −20°C.

### Characterizations of GSNO/RGZ@NPs

In order to characterize GSNO/RGZ@NPs, the final lyophilized products were redispersed in deionized water. The morphologies of NPs were observed by transmission electron microscope (TEM) (S-3400, Jeol, Tokyo, Japan). Particle size and distribution were measured using Malvern Particle Size Analyzer (Nano ZS, Malvern, Britain). The encapsulation efficiency (EE%) of GSNO and RGZ were calculated using the following formula:

(1) $$\text{EE}\%=C_{1}/C_{2}\times 100\%$$

*C*_1_ represented the concentration of drug encapsulated in the NPs, and *C*_2_ represented the total concentration of drug in the NPs.

The content of RGZ in NPs was analyzed using high-performance liquid chromatography (HPLC, Hitachi, Japan). The analysis method of RGZ was as follows: the mobile phase was a mixture of ammonium acetate solution (10 mM, pH 6.0) and acetonitrile (50:50, v/v). The column temperature was 35°C, and the flow rate was maintained at 1.0 ml/min. The injection volume was 20 μl, and UV detection was performed at 247 nm. Chromatographic separation was achieved by a reverse-phase Hypersil BDS C_18_ column (4.6 × 250 mm, 5 μm, Agilent Technologies, USA). The content of GSNO in NPs was measured using the Griess reagent kit and indicated the total NO release amount from NPs.

To evaluate the release profile of RGZ and GSNO from NPs, the freeze-dried products were reconstructed with 1 ml of deionized water and placed into the dialysis bag (10 kDa). The dialysis bags were placed in a beaker filled with 20 ml of PBS (pH 7.4) containing 0.5% SDS. The beakers were shaken at on shakers (37°C, 100 rpm/min). At predetermined time point, 1 ml of samples were removed and an equal amount of fresh released medium was supplied to maintain a constant volume. The amount of released RGZ was measured by HPLC. To figure out the release of NO from the NPs, Griess reagent kit was used and 10 ml PBS (pH 7.4) solution was chosen as release medium against the dialysis bag.

### Protective effect of GSNO/RGZ @ NPs on AGEs induced fibroblast injury

#### Preparation of AGEs-BSA and cell culture

Three grams of BSA and 0.9 g of D-glyceraldehyde were dissolved 100 ml of PBS (pH 7.2), followed by sterilization with 0.22 μm filter membrane and incubation at 37°C for 7 days. The obtained solution was dialyzed in PBS (pH 7.2) for 48 h to remove un-reactive D-glyceraldehyde. AGEs-BSA was obtained by freeze-drying the final solution.

#### Cell viability evaluation

The 3T3 cells were seeded in a 96 well plate at 2.5 × 10^4^ cells/well and incubated for 12 h. Different concentration of AGEs-BSA solutions prepared with culture medium were added into the plate (200 μl/well) to evaluate the deteriorated effect of AGEs. To evaluate the protective impact of GSNO/RGZ@NPs, the NPs with 0.1 μM, 1 μM or 10 μM of RGZ combined with AGEs-BSA containing medium were incubated with 3T3 cells. After 24 h, MTT solution and DMSO solution were added into the plates respectively under dark conditions. The absorbance of each well was detected by microplate reader at 570 nm and the survival rate of cells was calculated according to the following formula:

(2) $$\text{Cell viability}\;\left(\%\right)=\left({\text{OD}}_{t}-{\text{OD}}_{b}\right)/\left({\text{OD}}_{c}-{\text{OD}}_{b}\right)\times 100\%$$

The absorbance values of the test wells, blank wells and control wells were expressed as OD_t_, OD_b_ and OD_c_, respectively.

#### Anti-oxidative stress capacity

The 3T3 cells were seeded in a 12 well plate at 1 × 10^5^ cells/well and incubated for 12 h. The NPs containing 10 μM RGZ treated the cells against the 1 mg/ml of AGEs-BSA insulted cells. The 3T3 cells cultured by culture medium was used as negative control, while the ones treated with culture medium containing 1 mg/ml AGEs-BSA was used as positive control. To assess the level of intracellular ROS, the treated cells were stained by the DCFH-DA fluorescent dye and the DCF fluorescence labeled cells were analyzed using flow cytometry. SOD kits were used to test the level of intracellular SOD.

#### Inhibitory effect on cell apoptosis and changes of mitochondrial membrane potential

To assess the protective effect of NPs against the cell apoptosis induced by AGEs-BSA, the treated 3T3 cells were treated by the FITC labeled Annexin V and PI fluorescent dye and the fluorescence labeled cells were detected using flow cytometry. In similar, to investigate the changes of mitochondrial membrane potential induced by AGEs-BSA in 3T3 cells, 1 ml of JC-1 solution was added and the flow cytometry was used to sorting cells.

### Evaluation of inflammatory factor levels

The RAW264.7 cells were seeded in a 24 well plate at 5 × 10^4^ cells/well and incubated for 12 h. The cells were divided into five groups: control group (untreated group), lipopolysaccharide (LPS) group (treated with 5 μg/ml of LPS) and the NPs treated groups (treated with 5 μg/ml of LPS and NPs diluted solution at 50, 100 or 200 nM NO). After 24 or 48 h, the levels of IL-β, TNF-α and IL-6 were determined using enzyme-linked immunosorbent assay following the instructions of manufacturers.

### Preparation of NPs/hydrogel composite dressing

GSNO/RGZ@NPs were dissolved using 100 μl PBS (pH 7.4) and added 1 ml of 25 wt% Pluronic F127 solution under ice bath conditions. The solution was mixed well and placed at room temperature until forming into hydrogel.

### *In vivo* therapeutic efficacy of NPs/hydrogel compositive dressings

#### Establishment of wound model in diabetes rats

Streptozotocin (65 mg/kg) was injected intraperitoneally into SD rats (weighed 300–330 g). Three days later, the blood glucose level of each rat was confirmed with a blood glucose meter. One week after diabetes symptoms appeared in rats, the back of each rat was shaved and a 1.5 cm × 1.5 cm wound was fabricated on the back. After being divided into four groups, the rats were treated with 1 ml of PBS, F127 hydrogel, NPs suspension or NPs/hydrogel compositive dressing every two days.

#### Wound healing evaluation

The body weight and blood glucose levels of the rats in each group were determined during the treatment. The photos of the wounds on days 0, 2, 4, 6, 8, 10 and 12 post-treatment were taken and the wound areas were calculated using Image J software, respectively. On Day 6 and Day 12, the wound tissue was collected and subjected to histological stain (H&E, Immunohistochemistry and Masson staining). The wound healing rate was calculated using the following formula:

(3) $$\mathrm{Wound}\;\mathrm{closure}\;\mathrm{rate}\;(\%)=(S_{0}-S_{n})/S_{0}\times 100\%$$

*S*_0_ represented the area of the wound on Day 0 and *S_n_* represented the area of the wound at each time point.

### Hemolysis and cytotoxicity study

The NPs (GSNO/RGZ@NPs) were diluted to a series of concentrations of 10, 20, 50, 100 and 200 μM, respectively. About 500 μl of 4% erythrocyte suspension was added into the centrifuge tubes, to which 500 μl of different concentrations of the drug-carrying solutions were added, 500 μl of physiological saline was added into the negative control group and 500 μl of distilled water was added into the positive control group. After mixing, the above solutions were incubated at 37°C for 2 h, and then centrifuged (2000 r/min, 15 min) after 2 h. Photographs were taken to record the results. A small amount of the supernatant was measured in a 96-well plate, and the absorbance was determined at 540 nm to calculate the hemolysis rate.

### Statistical analysis

Data were shown as mean SD (standard deviation). Differences between groups were assessed using *t*-test and expressed as **** *P *<* *0.0001, *** *P *<* *0.0005, ** *P *<* *0.001 and ns, not significant.

### Ethics statement

All animal experiments were approved by the Animal Laboratory Ethics Committee of Shenyang Pharmaceutical University and performed under the guidelines for the care and use of laboratory animals (approval number: SYPU-IACUC-S2022-12-20-106).

## Results

### Characterization of GSNO/RGZ@NPs

**Figure 1.**
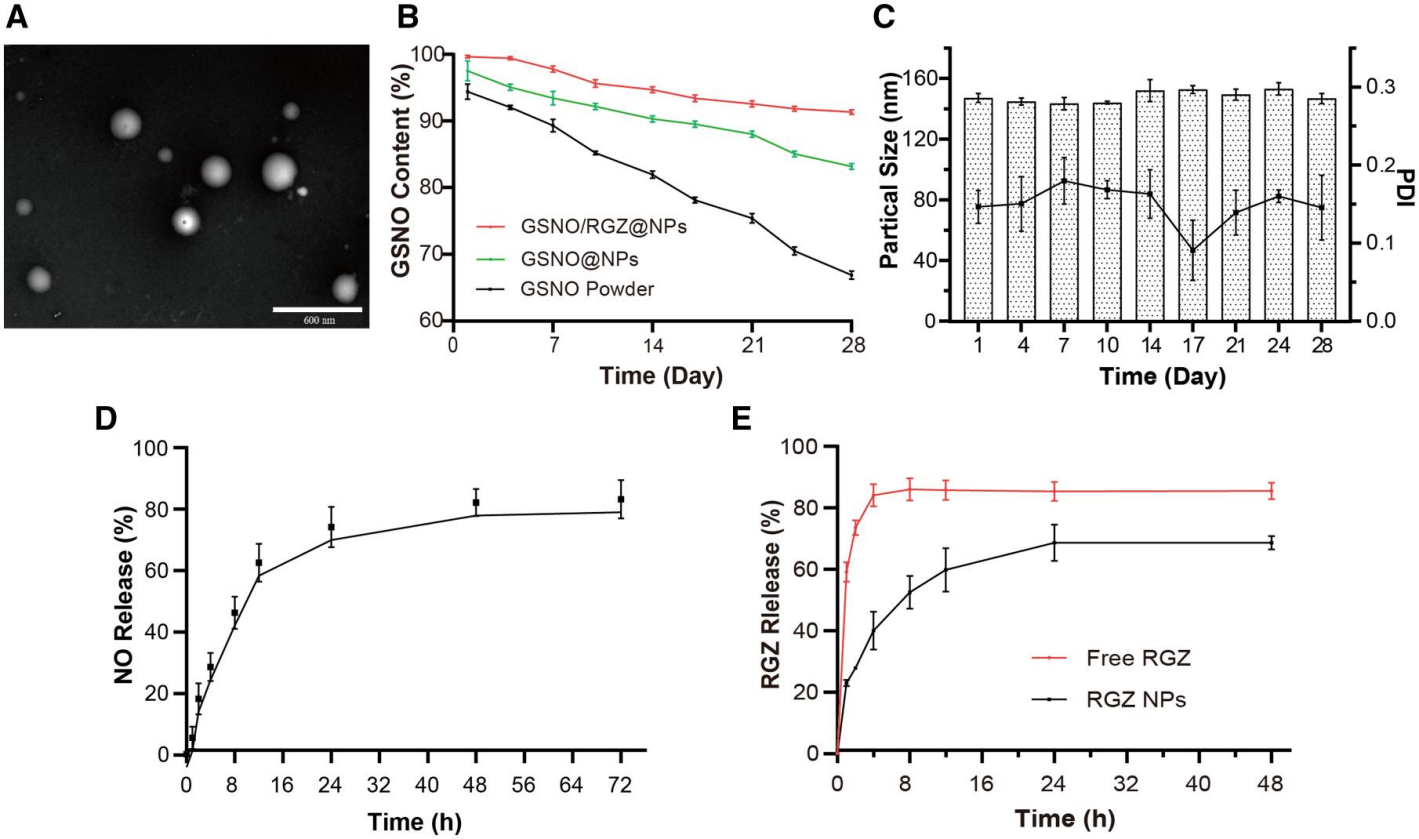
The performance of GSNO/RGZ@NPs. (**A**) TEM image for GSNO/RGZ co-loaded NPs; (**B** and **C**) Stability of NPs stored at 4°C under light-proof conditions after lyophilization; (**D** and **E**) cumulative release of NO and RGZ from GSNO/RGZ@NPs.

Table 1 showed the results of particle size, PDI and EE% of GSNO/RGZ@NPs. The PDI of the obtained NPs were all below 0.2, indicating that the particle size distribution was uniform. In order to confirm the effect of the lyophilization process on the particle size of our prepared NPs, we performed additional experiments (Table S1). The changes in particle size before and after lyophilization were examined by adding different amounts of RGZ within the organic phase. Some reduction in particle size can be seen after lyophilization (10 mg), but lyophilization has almost no effect on the particle size as the drug load is further increased (20 mg). TEM observation showed that the NPs were discrete and uniform spheres (Figure 1A).

**Table 1.** Characterizations of GSNO/RGZ@NPs

	Particle size (nm)	PDI	EE% (GSNO)	EE% (RGZ)
Before lyophilization	166.3 ± 5.4	0.189 ± 0.008	60.8 ± 2.1	64.9 ± 3.8%
After lyophilization	149.1 ± 5.8	0.146 ± 0.017		

Instability under heat and light can lead to the GSNO decomposition and premature NO release. The stability of drug loaded NPs was examined within 28 days under the condition of avoiding light at 4°C (Figure 1B). The content of GSNO encapsulated in NPs was significantly higher than that of in dry powder (83.2 ± 0.5% vs. 66.8 ± 0.6%), indicating that GSNO@NPs improved the stability of GSNO. The co-loading of GSNO and RGZ in NPs further improved the stability of GSNO as the GSNO content of the preparation only decreased by 8.6 ± 0.7% on Day 28. A possible reason for the increased stability of GSNO is the loading of RGZ into the organic phase, which results in the formation of a denser protective layer. During our experiments, we observed that the colostrum formed by loading RGZ into the organic phase was slightly more viscous than using the blank organic phase. During 28 days of storage, the particle size of GSNO/RGZ@NPs is relatively stable (148.1 ± 4.9 nm), and the particle size distribution is uniform (PDI < 0.2) (Figure 1C). The cumulative release amount of NO from NPs was about 80% within 48 h (Figure 1D). The cumulative drug release profile showed that free RGZ accumulated released more than 80% in the first 48 h, whereas the RGZ NPs released RGZ more slowly and only 65% released within 48 h (Figure 1E).

### Protective effects of GSNO/RGZ@NPs on AGEs induced fibroblast injury

**Figure 2.**
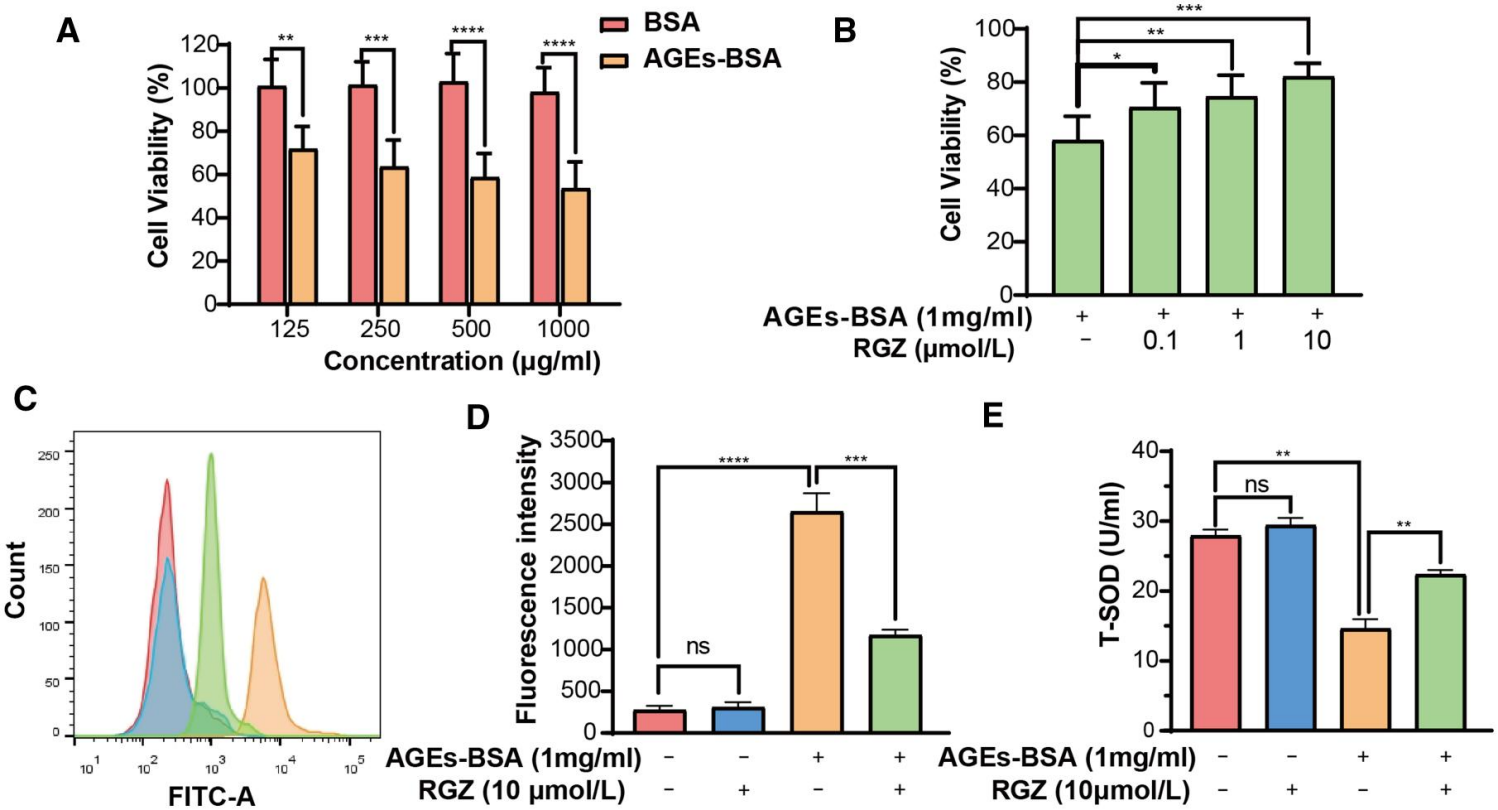
Protective effects of GSNO/RGZ@NPs on the cell inactivation and oxidative stress induced by AGEs. (**A**) Cytotoxicity caused by AGE-BSA at concentration of 125, 250, 500 and 1000 µg/ml. (**B**) GSNO/RGZ@NPs ameliorated AGEs-BSA-induced damages in 3T3 cells. (**C**) Fluorescence intensity of fibroblasts treated by different groups. (**D**) The value of mean fluorescence intensity. (**E**) The content of T-SOD in different groups. **** *P* < 0.0001, *** *P* < 0.0005, ** *P* < 0.001, ns, not significant (*n* = 3).

Fibroblasts play an important role in wound healing, but their functions may be inhibited by accumulated AGEs in diabetic wounds. To evaluate the cell damage mechanism induced by AGEs and the protective effects of GSNO/RGZ@NPs, cell viability, oxidative stress, apoptosis and mitochondrial membrane of 3T3 cells were investigated. AGEs-BSA was synthesized to simulate the accumulated AGEs in diabetes patients. We initially investigated the effect of different concentrations of AGEs-BSA on the survival of 3T3 cells. It could be seen that the toxicity effect of AGEs-BSA on 3T3 cells was concentration dependent (Figure 2A). The viability rate of 3T3 cells was only 53.6 ± 12.2% when the concentration of AGEs-BSA reached 1 mg/ml. GSNO/RGZ@NPs could inhibit the cytotoxicity caused by AGEs-BSA. As shown in Figure 2B, the survival rate of 3T3 cells increased to 82.1 ± 5.0% when the concentration of RGZ reached 10 μM.

The capability of the obtained NPs to reverse the imbalance of cellular oxidative stress induced by AGEs-BSA was evaluated by measuring the expression levels of reactive oxygen species (ROS) and superoxide dismutase (SOD) in 3T3 cells. The results of Figure 2C and D displayed the intracellular ROS level by measuring of DCF molecular fluorescence intensity. The ROS level of 3T3 cells was obviously increased after treatment with AGEs-BSA (*P *<* *0.0001). Addition of GSNO/RGZ@NPs alleviated the oxidative stress since the ROS level in the NPs treated group was significantly reduced compared to AGEs-BSA group (*P *<* *0.0005). Similar results were also observed in the expression levels of SOD (Figure 2E). These results indicated GSNO/RGZ@NPs were beneficial for the recovery of oxidative stress levels of 3T3 cells.

**Figure 3.**
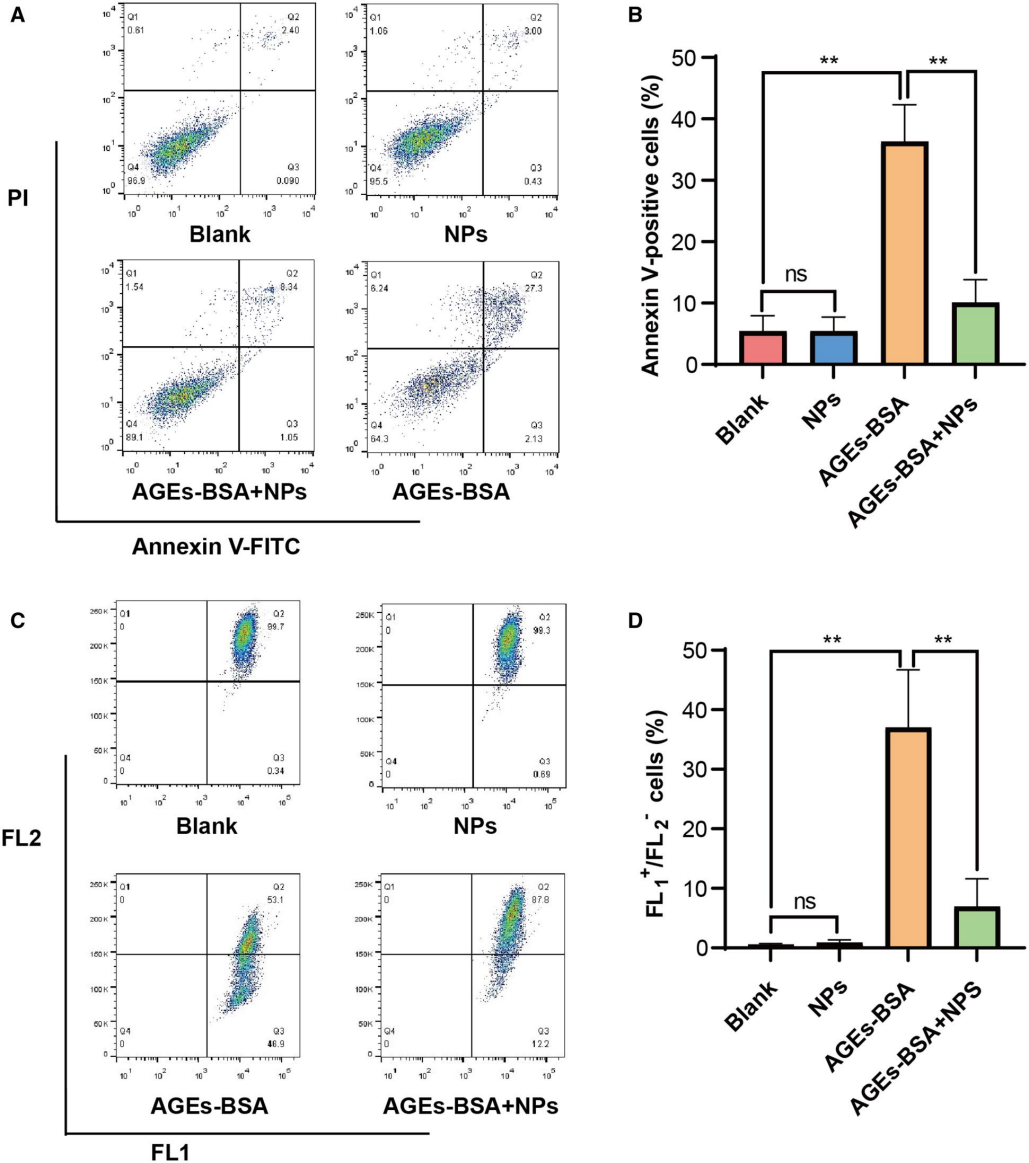
Inhibitory effect of GSNO/RGZ@NPs on cell apoptosis and changes of mitochondrial membrane potential induced by AGEs-BSA. (**A**) Representative flow cytometry scatter plot images of annexin V-FITC/PI staining. (**B**) The statistics graph of annexin V-positive cells. (**C**) Representative flow cytometry scatter plot images of JC-1 staining. (**D**) The statistics graph of ${\text{FL}}_{1}^{+}$/FL_2_ cells. ** *P* < 0.001, ns, not significant（*n* = 3）.

Annexin V-FITC/PI double staining method was used to examine the protective effect of GSNO/RGZ@NPs on AGEs-BSA induced cell apoptosis. As shown in Figure 3A, existence of apoptotic cells population further confirmed the cytotoxic effect of AGEs. Percentage of Annexin V-positive cells significantly reduced after the treatment with NPs, indicating GSNO/RGZ@NPs could inhibit the apoptosis of 3T3 cells induced by AGEs.

Mitochondrial dysfunction is closely related to the occurrence and development of diabetes [34]. Mitochondrial membrane potential depolarization is as an important early signal of cell apoptosis. JC-1 staining method was used to detect the changes of the cell mitochondrial membrane potential. As shown in Figure 3C and D, cell population shifted from red fluorescence to green fluorescence when treated with AGEs-BSA, and the ratio of ${\text{FL}}_{1}^{+}$ and ${\text{FL}}_{2}^{-}$ cells substantially higher than the untreated group (*P *<* *0.001), indicating that AGEs-BSA was related to early apoptosis of 3T3 cells. Compared with the AGEs-BSA group, the cell population of the NPs treated group shifted towards the red fluorescence region, and the ratio of ${\text{FL}}_{1}^{+}$ and ${\text{FL}}_{2}^{-}$ cells significantly reduced indicating GSNO/RGZ@NPs prevented cell apoptosis induced by AGEs.

### Anti-inflammatory effect of GSNO/RGZ@NPs

**Figure 4.**
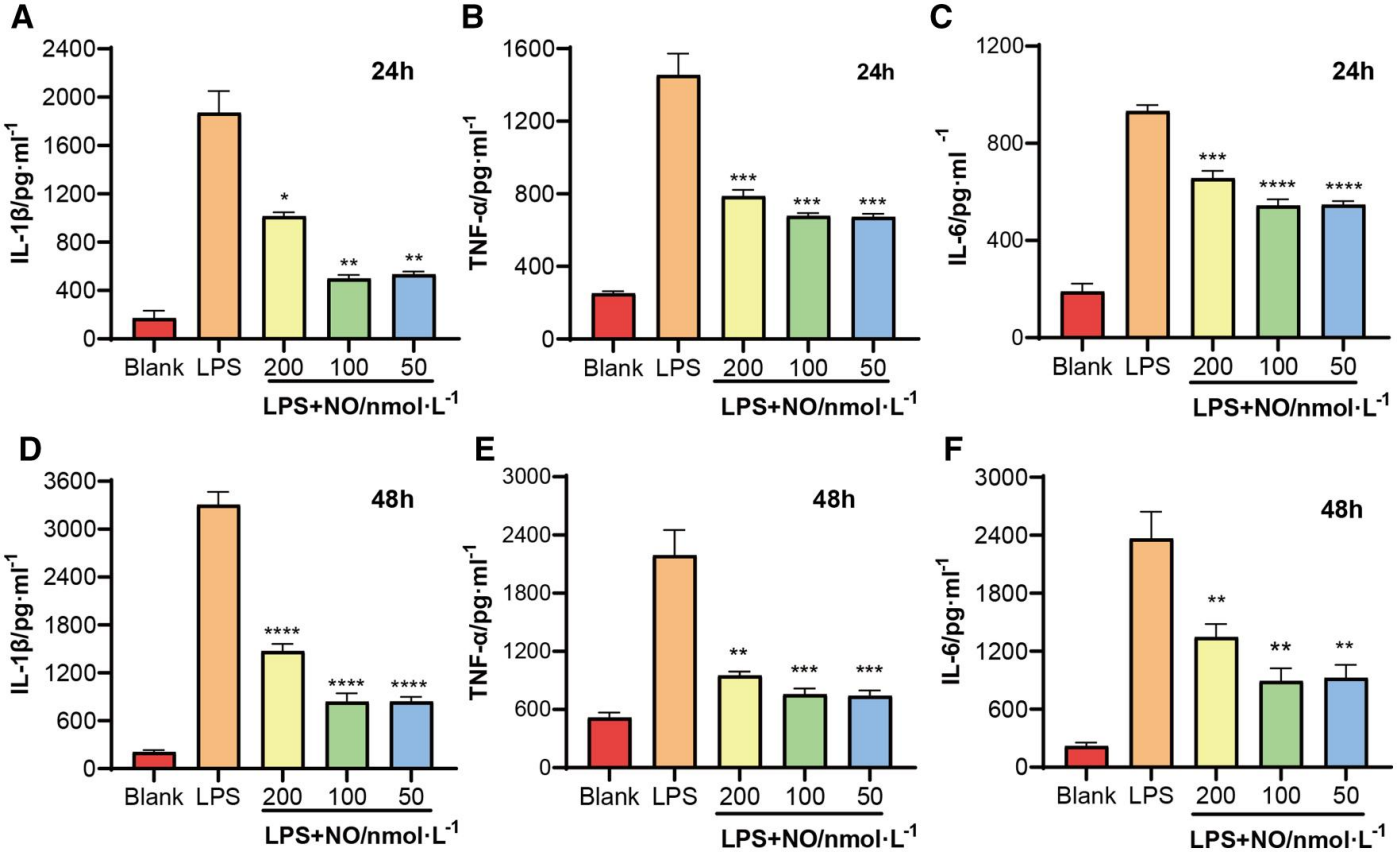
Effects of GSNO/RGZ@NPs containing different concentrations of NO on the expression of inflammatory factor. The levels of (**A** and **D**) IL-β, (**B** and **E**) TNF-α and (**C** and **F**) IL-6 in RAW264.7 at 24 and 48 h *in vitro*.

AGEs are also be considered to promote macrophage polarization towards pro-inflammatory phenotype. Therefore, we also confirmed NPs were able to inhibit M1 polarization by evaluating macrophage inflammatory factor expression. The level of pro-inflammatory factors treated with NPs dilutions containing different concentrations of NO were investigated and the results were shown in Figure 4. Compared with the control group, the expression of inflammatory factors in RAW246.7 cells induced by LPS significantly increased, indicating the successful establishment of the cellular inflammatory model. The level of IL-β, TNF-α and IL-6 were all down-regulated in NPs groups at 24 and 48 h after treatment. Notably, cells in the 200 nM group expressed the highest levels of inflammatory factors among all the NPs treated groups. It revealed that NPs loaded NO could alleviate wound inflammation, however the high level of NO did not provide the superior effect than those in relative lower levels.

### *In vivo* therapeutic efficacy of NPs/hydrogel compound dressings

**Figure 5.**
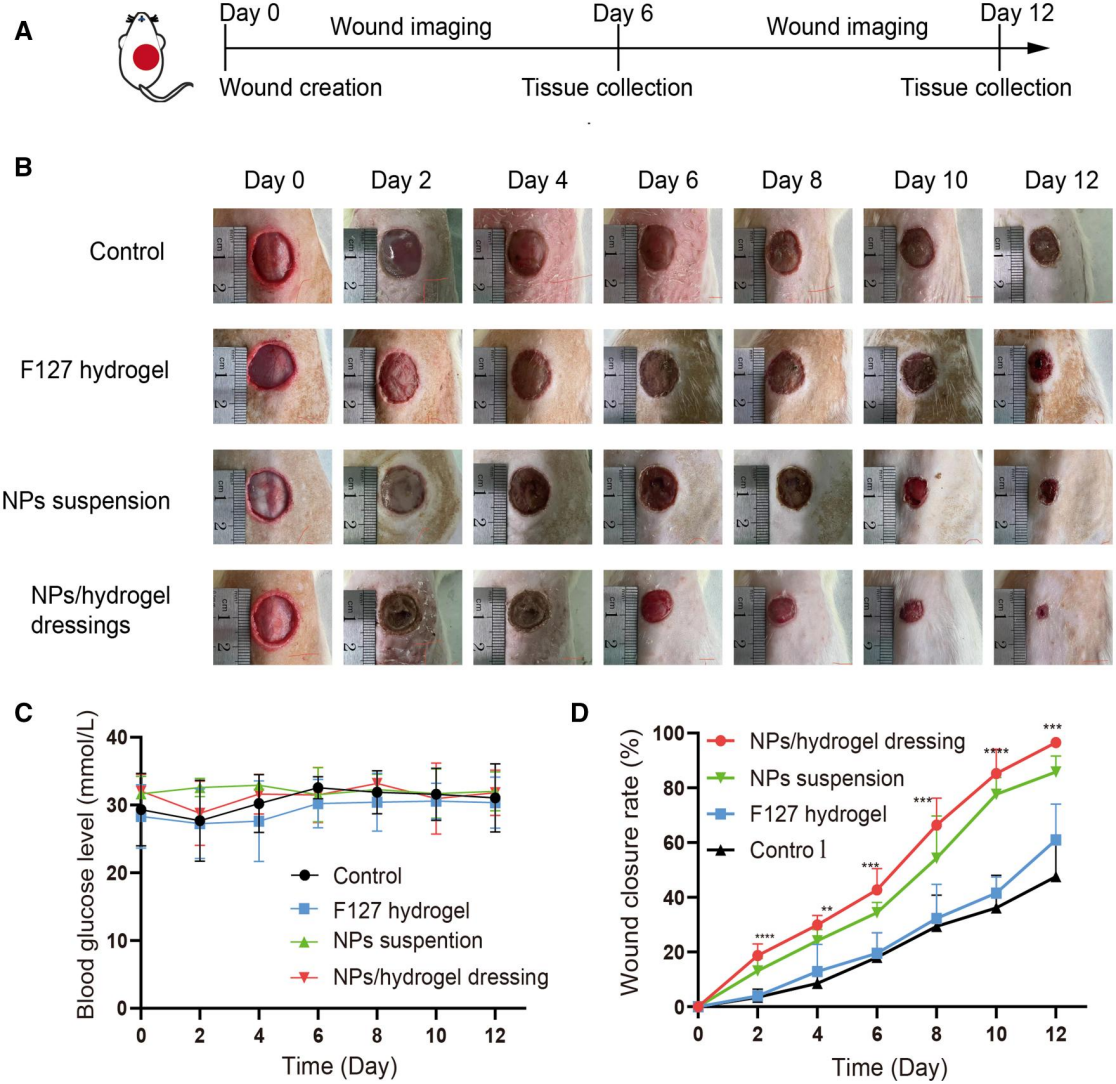
*In vivo* diabetic wound healing evaluation. (**A**) Schematic illustration of the design of animal experiments. (**B**) Representative photographs of wound changes during 12 days. (**C**) Changes of blood glucose level; (**D**) Wound closure rate curve in different groups. **** *P *<* *0.0001, *** *P *<* *0.0005, ** *P *<* *0.001 (*n* = 5).

A diabetic wound model was employed to assess the *in vivo* therapeutic effect produced by the prepared NPs and NPs/hydrogel composite dressing. As shown in Figure 5C, all rats were induced into hyperglycemic states. The blood glucose levels of each group were all higher than 20 mM during the administration period, indicating that the diabetes model was successfully established. Afterwards, a 1.5 cm × 1.5 cm wound was fabricated on the back after 1 week when rats displayed diabetic symptoms. The wound area was monitored every 2 days and the representative photographs of skin wound healing was shown in Figure 5B. The wound closure rate in the control group was less than 50% on the 12th day (Figure 5D). The group of F127 hydrogel improved the wound healing rate compared with the control group, but the ability was very limited (61.0% ± 13.1% vs. 47.6% ± 13.7%). More importantly, the wound of diabetes rats healed almost completely on Day 12 for the NPs suspension and the NPs/hydrogel composite dressings groups respectively (96.5% ± 1.9% and 87.7 ± 5.8%).

**Figure 6.**
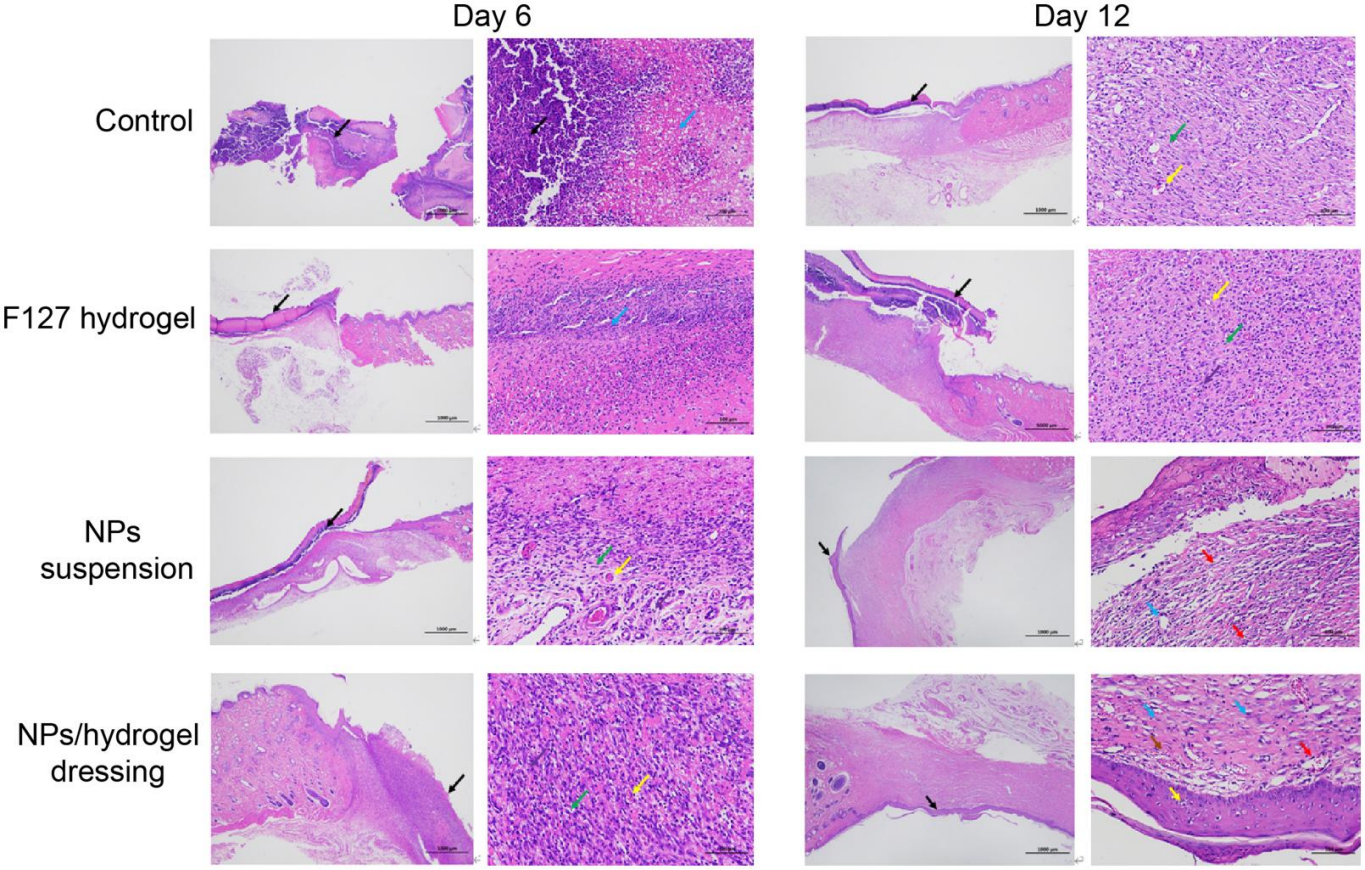
Representative H&E-stained histopathological images on wound healing under different treatment at Day 6 and Day 12.

HE staining was used to further characterize the histopathology manifestations of each group during the wound healing process. According to the H&E staining results on Day 6 (Figure 6), a large areas of skin necrosis were observed in the untreated and F127 groups (black arrows indicating pyknosis and the blue arrows pointing to necrosis debris). No fibrous cells or collagen fibers appeared in both of groups. Reversely, for the NPs suspension and the NPs/gel groups, there was a large number of fibroblasts appeared in the deep dermis (green arrow). Angiogenesis of capillaries was also observed (yellow arrow). On Day 12, large areas of skin necrosis (the black arrow) with significant inflammatory cell infiltration (purple arrow) were still presented in the untreated and gel groups. In contrast, the other two groups generated new epidermal layer and large numbers of fibroblasts (red arrow) and neovascularization (yellow arrows) were also observed.

**Figure 7.**
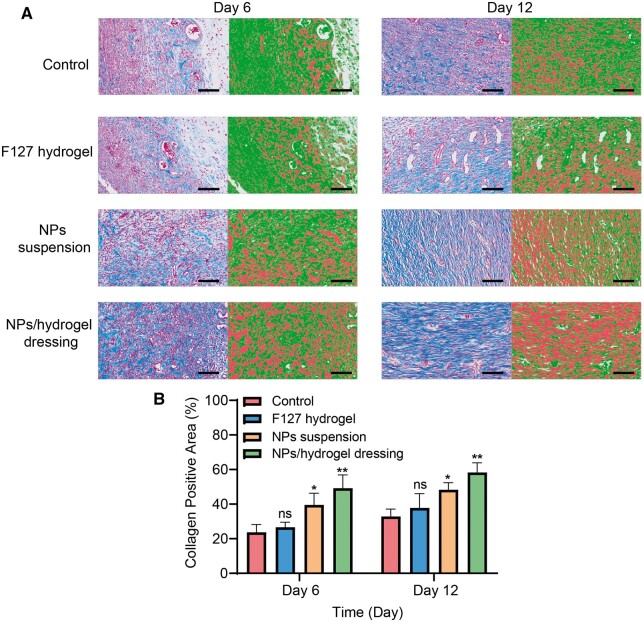
Results of collagen deposition from different treatment. (**A**) Masson staining images of the diabetic wounds on Day 6 and Day 12. Scale bar, 100 µm. (**B**) The statistics graph of collagen positive area (%). Compared with control group: ns, not significant, * *P *<* *0.05, ** *P *<* *0.001 (*n* = 3).

During the stages of cell proliferation and tissue regeneration, collagen fibers play a crucial role in the remodeling of ECM [35]. Therefore, Masson staining was performed to further analyze the wound healing process of each group by examining the collagen deposition (Figure 7A and B). Increased collagen synthesis was observed in the NPs suspension and the NPs/hydrogel compositive dressings treated groups. The collagen fibers of group treated with NPs/hydrogel compositive dressings intertwined and formed a dense network structure on Day 12. Also, this group owned the highest percentage of collagen positive area (49.2 ± 7.7% on Day 6 and 58.3 ± 5.6% on Day 12). All of the results suggested NPs/gel compositive dressings could improve ECM remodeling and tissue regeneration by enhancing collagen synthesis.

**Figure 8.**
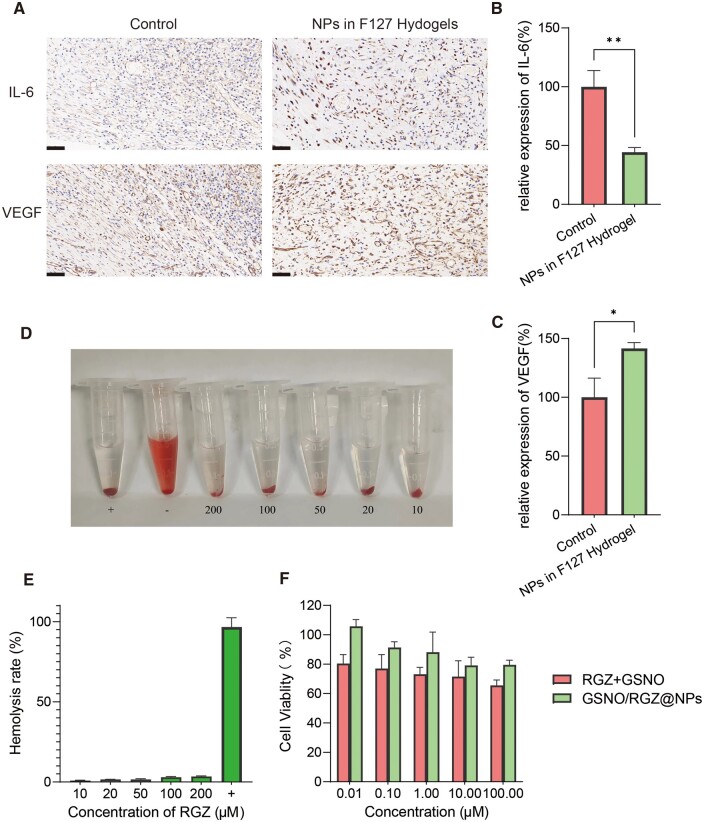
Results of immunostaining analysis, cytotoxicity and hemolysis experiments. (**A**) Immunohistochemical photographs of control and gel groups. Scale bar, 50 µm. The inflammatory cytokine IL-6 (**B**) and TNF-α (**C**) alternation of diabetic wounds after different treatments. Compared with control group: * *P *<* *0.05, ** *P *<* *0.001 (*n* = 3). (**D**) Digital images of RBCs after being treated with NPs. (**E**) Hemolysis percentage of NPs compared with positive control. (**F**) The cell viability of 3T3 cells from different treatment.

Figure 8A showed immunohistochemical photographs of inflammation-causing and inflammation-suppressing factors in the control and gel groups, and Figure 8B and C were the measured *in vivo* expression levels of inflammatory factors, respectively. Compared with the control group, the gel group significantly reduced IL-6 levels and elevated VEGF levels in diabetic rat wounds, further demonstrating that the gel was able to inhibit the inflammatory response in diabetic wounds. Hemolysis and cytotoxicity were also evaluated for GSNO/RGZ@NPs. Figure 8D showed that co clearly visible hemolysis on RBC were found for NPs. Quantitative data on hemolysis displayed the hemolysis of NPs was very low, and the hemolysis rate remained below the hemolysis threshold (<5%) even for the NPs with the highest concentration of RGZ (200 μM). The results of cytotoxicity experiments showed that the cell survival rate of free drugs at 100 μM was already lower than 80%. In contrast, the cell survival rate after loading the drug into the NPs remained higher than 80%, which indicated that the prepared GSNO/RGZ@NPs could improve the cytotoxicity of the drugs.

## Discussion

The toxic effect of AGEs is a main mechanism of refractory diabetic wound [9, 36, 37]. AGEs change the structure and function of cells via cross-linking and multiple signaling pathways involved the surface receptors like RAGEs, as shown in Scheme 1. The activation of these important signaling pathways leads to the generation of intracellular ROS, upregulation of inducible NO synthase (iNOS) expression, downregulation of endothelial NO synthase (eNOS) expression and transcription of pro-inflammatory genes, which ultimately results in inflammation and cell apoptosis. The development of new therapeutic strategies based on AGEs targets will have tremendous clinical implications.

Current works on AGEs solution strategy have included applying AGEs or its receptor inhibitors, blocking of AGEs-RAGEs signaling pathways and *in situ* removal of AGEs. Sun *et al.* [38] employed a metal-organic frameworks as a dual proangiogenic drug delivery system for targeting RAGE signaling pathway. The authors showed that a RAGE inhibitor released from the framework reduced inflammation-induced impairment of angiogenesis by promoting M2 macrophage polarization. Moreover, the continuous release of cobalt ions further improves angiogenesis at the wound site directly via activation of HIF-1α. Based on anti-glycosylation effect of CeO_2_ nanoenzyme, an OCE hydrogels with antioxidant and antiglycation properties were obtained by doping CeO_2_ nanorods into a crosslinked network via Schiff base reaction [39]. This gel excipient has been proved to own dual functions of inhibiting AGE accumulation and scavenging ROS. Recently, a novel vRAGE-ELP fusion protein has been designed to inhibit AGE-RAGE mediated signaling pathways [12]. In this study, vRAGE, was engineered in the form of fusion proteins to provide a stable method of protein delivery in highly protein-hydrolyzed wound environments. One Dose of vRAGE-ELP treatment effectively accelerated wound healing in diabetic mice. *In situ* removal of AGEs from diabetic wounds using peptide-modified chiral dressing was developed to effectively achieve timely revascularization and accelerate wound healing [40]. The dressing consists of L-phenylalanine and cationic hexapeptide co-assembled to form helical nanofibers, which are later cross-linked with hyaluronic acid through hydrogen bonding. The abundance of chiral sites contained in the hydrogel dressing enabled *in situ* removal of AGEs through stereoselective interaction of AGEs with chiral sites. In this study, our prepared NPs/hydrogel composite dressings showed that local co-delivery of RGZ and GSNO could inhibits AGE-induced inflammation, stimulating collagen fiber formation and angiogenesis. We also analyzed the potential mechanism of the combined therapeutic strategy in inhibiting the toxic effects of AGEs from the following aspects.

Accumulation of AGEs leads to increase of abnormal cell apoptosis, which negatively impacts wound healing [41, 42]. Fibroblasts are important for the production of collagen and new ECM during the wound healing process. AGEs induced fibroblast apoptosis through different signaling pathways, such as ROS and mitochondrial pathways [43, 44]. Increased apoptosis of fibroblasts was also proved to impair wound healing of diabetes rats [45]. To confirm the protection of the designed co-delivery system against fibroblast apoptosis, we investigated the effect of GSNO/RGZ@NPs on fibroblasts survival in an AGEs environment. The prepared NPs inhibited the apoptosis and alleviated the polarization of mitochondrial membrane potential of 3T3 cells induced by AGEs-BSA (Figure 3A and B). Accumulating level of AGEs also leads to increased intracellular ROS, which induces the oxidative stress, and ultimately causing inflammation and cell apoptosis [46, 47]. Thus, the protective effect of NPs against cellular oxidative stress was also been examined as shown in Figure 3D and E. Our results showed that the level of ROS in 3T3 cells treated with co-loaded NPs was significantly lower than that treated with AGE-BSA. SOD is an antioxidant enzyme which plays an important role in cellular antioxidant balance. It protects cells from oxidative damage by scavenging superoxide anion free radicals in mitochondria [48, 49]. The intracellular SOD level in the NPs group was higher than that in the AGEs-BSA group. All the results indicated that the co-delivery NPs could reverse the toxic effects of AGEs.

Furthermore, we investigated the impact of co-delivery NPs on reducing the inflammatory response. Persistent inflammation at the wound bed of DFU is an important reason to hinder the formation of healthy tissue [50]. An important effect of AGEs/RAGEs interaction is activating pro-inflammatory transcription factor, such as NF‐κB [51], which is associated with upregulation of numerous inflammatory cytokine expression [9]. The chronic wound infiltrated by macrophages leaded to a dramatic increase of pro-inflammatory cytokines [52]. Therefore, the anti-inflammatory effect of prepared NPs on macrophages was assessed. The level of IL-β,TNF-α and IL-6 were all down-regulated in the NPs groups, indicating that co-delivery of NPs could limit the inflammatory response. This anti-inflammatory effect may come from the local release of NO since the ability of NO to regulate inflammation at the wound site had been widely demonstrated [53, 54]. However, the effect might be concentration-limited, with only ∼nM level of NO producing anti-inflammatory processes (Figure 4) [55].

Current evidence suggested that AGEs induced upregulation of iNOS expression and downregulation of eNOS expression. Low concentration physiological NO produced by eNOS was helpful for the proliferation of endothelial cells, collagen deposition and angiogenesis [56]. While high concentration irritability NO produced by iNOS had a negative effect on wound healing due to the nitro-oxidative stress pro-inflammatory pathways [57]. Along with the downregulation of eNOS, the NO level of diabetes wound fluid was significantly lower than that of healthy wound fluid [58]. Accordingly, supplementing exogenous NO was beneficial for reversing the negative effects of AGEs. In this study, GSNO was selected as an endogenously NO donor and co-loaded with RGZ in NPs to improve the stability and the kinetics of NO release. Notably, NPs also improved the insufficient penetration of NO in the wound bed. In a previous work, the wound closure rate was less than 70% on the 21st day when topical application of GSNO-containing hydrogel [59]. For our designed co-delivery system, the wound healing rate reached over 90% on day 12. Therefore, the use of combined therapeutic strategy with a pluripotent role may be a potential in the treatment of DFU.

## Conclusion

In this study, we demonstrated that a combined therapeutic strategy aimed at inhibiting the toxic effects of AGEs was benefit for accelerating diabetic wound closure. The NPs/hydrogel composite dressings blocked the harmful effects of AGEs in many ways: it augmented the survival of fibroblasts, reduced oxidative stress levels, reversed the change of mitochondrial membrane potential, and decreased the proinflammatory cytokine expression. This local co-delivery system displayed multiple therapeutic effects, including decreasing tissue inflammation, and stimulating collagen fiber formation and angiogenesis. The NPs/hydrogel composite dressing offers a new way for the treatment of chronic diabetic wounds.

## Supplementary Material

rbae062_Supplementary_Data
