# Supplementary material for: Combined therapeutic strategy based on blocking the deleterious effects of AGEs for accelerating diabetic wound healing
Source: Regen Biomater. 2024 Jun 5;11:rbae062. doi: 10.1093/rb/rbae062 (PMC11424028; doi:10.1093/rb/rbae062)
Supplement: rbae062_Supplementary_Data [file rbae062_supplementary_data.docx]

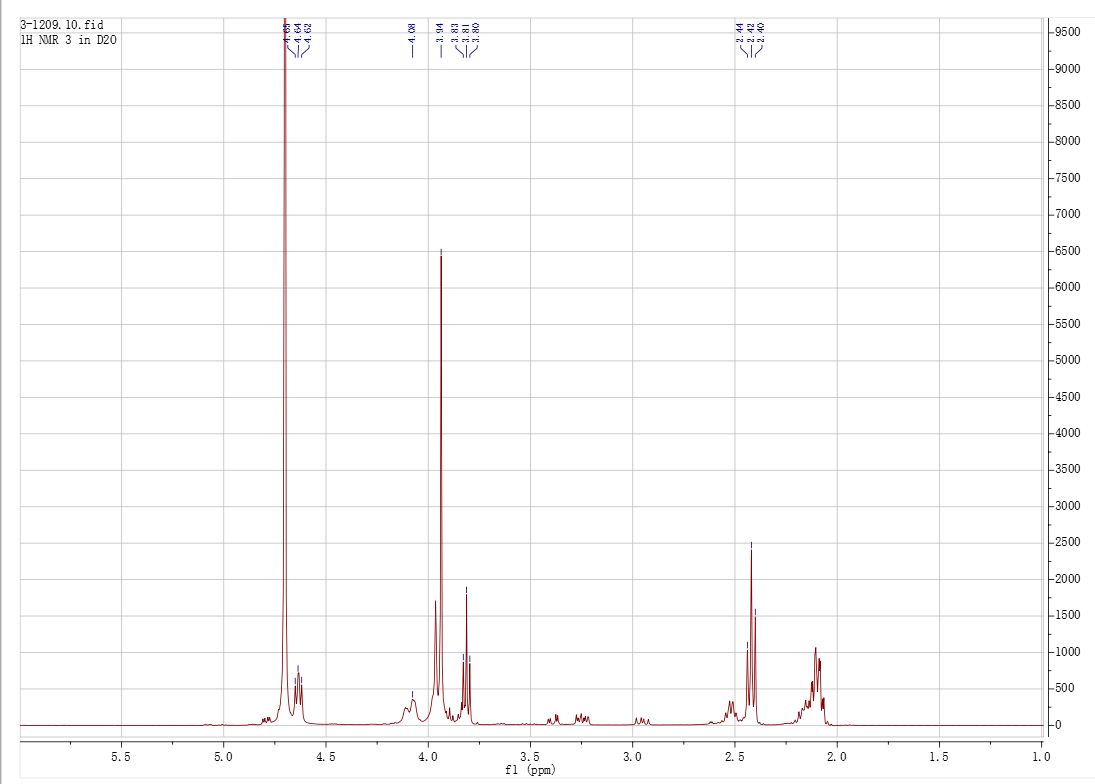


Figure S1. The functional groups of GSNO were attributed by 400 M 1H NMR with D2O as solvent. The NMR hydrogen spectra of GSNO were analyzed as follows:1H NMR (400 MHz, D2O): δ 4.63 (app.t, 1 H), 4.08 (dd, 2 H), 3.94 (s, 2 H), 3.81 (t, 1 H), 2.42 (t, 2 H), 2.08 (app.nonet , 2 H),.


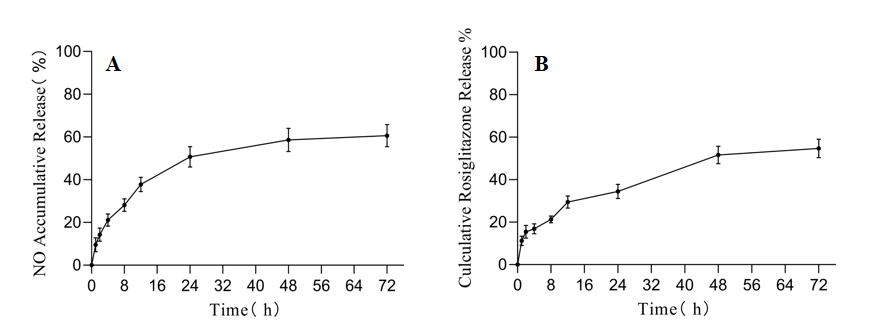


Fig. S2 Cumulative release of (A) NO, and (B) RGZ from GSNO/RGZ@NPs in F127 Hydrogels（n=3）

TableS1 Effect of different RGZ dosage to polymer on the diameter and PDI of GSNO/RGZ@NPs.

|  |  | Partical Size（nm） | PDI |
| --- | --- | --- | --- |
| 10 mg | before lyophilization | 166.3±5.4 | 0.189±0.008 |
|  | after lyophilization | 149.1±5.8 | 0.146±0.017 |
| 20 mg | before lyophilization | 199.3±11.7 | 0.180±0.024 |
|  | after lyophilization | 200.1±2.7 | 0.137±0.048 |
